# Pathway Analysis of Allulose as a Sugar Substitute in Mitigating Thrombotic Risks in Sickle Cell Disease Patients

**DOI:** 10.3390/nu16244295

**Published:** 2024-12-12

**Authors:** Seong Su Choi, Eun Ji Kim, Su-Kyung Shin, Ji-Yoon Lee, Ji Won Han, Eun-Young Kwon, Heekyong R. Bae

**Affiliations:** 1 Department of Food Science and Nutrition, Kyungpook National University, 80 Daehak-ro, Buk-ku, Daegu 41566, Republic of Korea; knu19css@gmail.com (S.S.C.); meungzzi@naver.com (E.J.K.); kitty49355255@gmail.com (J.-Y.L.);; 2 Center for Food and Nutritional Genomics, Kyungpook National University, Daegu 41566, Republic of Korea; 3 Center for Beautiful Aging, Kyungpook National University, Daegu 41566, Republic of Korea

**Keywords:** allulose, erythritol, mitochondrial dysfunction, sickle cell disease

## Abstract

Long-term consumption of erythritol, a widely used sugar substitute, has been associated with increased risks of thrombosis and cardiometabolic diseases. In this study, we investigated the effects and mechanisms of allulose in mitigating these risks compared to erythritol using the clusterProfiler tool in R (version 4.12.6). Since a high-fat diet (HFD) is known to enhance platelet aggregation, we compared the pathways related to these processes between groups of mice treated with allulose and those treated with erythritol. While erythritol exacerbated HFD-induced increased platelet aggregation, allulose treatment significantly reduced it. Further analysis of platelet gene expression in sickle cell disease (SCD) patients to explore the potential of using sugar substitutes revealed that platelet coagulation mechanisms could be exacerbated by HFD. Additionally, the top up- and downregulated pathways in SCD were significantly reduced in the allulose-treated group compared to the erythritol group. Specific mechanisms related to this include the mitochondrial complex I and mitochondrial translational process as potential pathological factors in platelet coagulation related to SCD. Therefore, this study demonstrates that allulose may offer a safer alternative to erythritol in dietary applications, especially in individuals susceptible to thrombotic events, by modulating critical pathways associated with platelet function and mitochondrial activity.

## 1. Introduction

The growing use of sugar substitutes has raised concerns regarding their effects on cardiovascular health, with circulating erythritol being suggested as a potential marker for predicting metabolic risks [1]. Erythritol, one of the most popular sugar substitutes, has been widely adopted due to its low caloric content and minimal effect on blood sugar levels. However, recent studies have suggested that erythritol may increase the risk of thrombosis, a serious condition where blood clots form and block blood vessels, leading to complications such as stroke and heart attacks [2]. This concern is especially relevant for individuals with SCD, who are already predisposed to abnormal platelet activation and frequent thrombotic events [3]. Consequently, it is critical to explore safer sugar alternatives that do not exacerbate these risks.

Allulose is a rare monosaccharide with a chemical structure similar to fructose, making it about 70% as sweet as sucrose but with nearly zero calories [4,5]. Its molecular configuration differs slightly from fructose, which prevents it from being metabolized efficiently by the body, contributing to its low caloric content. Biologically, allulose has shown potential benefits, such as improving insulin sensitivity, lowering blood glucose levels, and reducing fat accumulation [6,7]. Early studies also suggest that it may have fewer negative effects on metabolic and cardiovascular health compared to other sugar substitutes, as it appears to modulate inflammation and oxidative stress, both of which are linked to metabolic disorders and cardiovascular risk [8,9,10]. However, its potential effects on thrombosis and platelet function in high-risk populations like SCD patients remain poorly understood. Particularly, while sugar substitutes are often assessed for their metabolic and glycemic impacts, their influence on liver function and subsequent effects on thrombosis have not been fully explored.

Chronic inflammation, particularly triggered by a HFD, plays a pivotal role in increasing the risk of thrombotic events [11,12]. HFD induces metabolic dysfunction, leading to the accumulation of lipids in the liver, which in turn promotes inflammatory cytokine production such as tumor necrosis factor-α (TNF-α) and interleukin-6 (IL-6) [13]. These cytokines drive systemic inflammation, which disrupts normal vascular function and enhances platelet activation [14,15]. This heightened platelet activity increases the likelihood of aggregation and clot formation, thereby elevating the risk of thrombosis. By modulating liver function through dietary interventions like allulose, there is potential to reduce this systemic inflammatory response.

Further, mitochondrial dysfunction in the liver has been associated with oxidative stress and metabolic disturbances, which are known contributors to platelet dysfunction and thrombosis [16,17]. Allulose has shown promise in enhancing mitochondrial function, which may reduce oxidative stress and improve overall metabolic health, indirectly influencing platelet activity [8]. The potential for allulose to modulate metabolic pathways in the liver, reduce inflammation, and enhance mitochondrial function presents a compelling case for its role in mitigating thrombotic risks, particularly when compared to erythritol.

This study aims to investigate whether allulose can offer antithrombotic benefits by modulating liver function and related systemic pathways. Using gene expression analysis, we compared the effects of allulose and erythritol on liver tissue in mice subjected to a HFD, focusing on pathways involved in platelet activation, coagulation, and mitochondrial function. Additionally, to assess the relevance of these findings in a high-risk population, we analyzed platelet gene expression data from SCD patients, who are particularly prone to thrombotic complications. By comparing these two sets of data, we aim to provide insights into the potential of allulose as a safer sugar substitute that reduces the risk of thrombosis.

## 2. Materials and Methods

### 2.1. Animal Experiments

All animal experiments were conducted following the approval of the Animal Ethics Committee (KNU 2022-0458), granted on 23 November 2022. For the study, 32 male C57BL/6J mice, 4 weeks old, were acquired from JA BIO (Suwon, Republic of Korea). The mice were housed individually in a controlled environment maintained at 22 ± 2 °C with a 12 h light-dark cycle. After a one-week acclimation period, the mice were randomly divided into four groups (*n* = 8 per group), ensuring minimal variation in body weight between groups. These groups included a normal diet group (ND, 10% calories from fat), a high-fat diet group (HFD, 40% calories from fat), a high-fat diet supplemented with 5% allulose (ALLU), and a high-fat diet supplemented with 5% erythritol (ERY). The animals were fed their respective diets for 16 weeks, with weekly monitoring of body weight and food intake. As we previously reported [7], the baseline body weight of mice in each group showed no significant differences. After the period of 16 weeks, ALLU group exhibited the lowest body weight gain, with a statistically significant difference. The HFD and ERY group showed a significantly higher body weight gain compared to the ND group. Food intake was similar in all groups. Energy intake was significantly higher in the high-fat diet consumption group. The ALLU group showed lowest food efficiency ratio, while HFD and ERY group exhibited significantly higher energy efficiency compared to the ND group. The detailed data will be provided in a separate publication.

### 2.2. Library Construction and RNA Sequencing

At the end of the 16-week feeding period, tissues were collected, and total RNA was extracted from liver samples using TRIzol reagent. The isolated RNA was stored in a 75% ethanol solution. RNA concentration was determined with Quant-IT RiboGreen (Invitrogen, Waltham, MA, USA #R11490), and the RNA quality was verified using a TapeStation RNA screentape (Agilent, Santa Clara, CA, USA #5067-5576), ensuring an RNA integrity number (RIN) of greater than 7.0. For further analysis, three individuals were randomly selected from each group. The RNA library was constructed using high-quality RNA, and total RNA was refined using the Ribo-zero rRNA removal kit. Library preparation was performed with 1 µg of total RNA using the Illumina TruSeq Stranded mRNA Sample Prep Kit (Illumina, San Diego, CA, USA #20020595). Poly-A mRNA was purified using poly-T magnetic beads, followed by fragmentation of mRNA into smaller fragments with divalent cations at elevated temperatures. First-strand cDNA synthesis was performed using SuperScript II reverse transcriptase (Invitrogen, Waltham, MA, USA #18064014), and second-strand synthesis was conducted using DNA Polymerase I, RNase H, and dUTP. The cDNA fragments underwent end-repair, adapter ligation, and PCR enrichment to generate the final cDNA library. The cDNA libraries were quantified using KAPA Library Quantification kits (KAPA BIOSYSTEMS, Wilmington, MA, USA #KK4854) and quality-assessed with the TapeStation D1000 ScreenTape (Agilent Technologies, Santa Clara, CA, USA #5067-5582). Indexed libraries were sequenced on an Illumina NovaSeq platform (Illumina, Inc., San Diego, CA, USA), providing paired-end (2 × 100 bp) transcriptome sequencing for 20 samples. Following sequencing, pre-processing steps were applied to remove adapter sequences, contaminant DNA, and PCR duplicates to reduce bias. Reads were aligned to the reference genome using HISAT2 (version 2.1.0), and transcript assembly was conducted using StringTie (version 2.1.3b). Gene expression profiles were quantified using FPKM (Fragments Per Kilobase of transcript per Million mapped reads) and TPM (Transcripts Per Kilobase Million) values. Differentially expressed genes (DEGs) were identified with DESeq2, with criteria set at |log2 fold change| ≥ 2 and a raw *p*-value < 0.05 based on nbinomWaldTest.

### 2.3. SCD Patient Data Collection

Gene expression data focusing on platelet gene expression in SCD were obtained from the Gene Expression Omnibus (GEO) (accession number GSE11524), comprising 18 SCD samples and 12 healthy control samples from African American volunteers. The gene expression profiles were generated using the Affymetrix Human Genome U133 Plus 2.0 Array platform. Differences in platelet gene expression between SCD patients and healthy controls were analyzed using the GEO2R tool (7th March 2024), allowing for the comparison of normalized expression levels between the two groups.

### 2.4. Pre-Ranked Gene Set Enrichment Analysis (GSEA)

Pre-ranked Gene Set Enrichment Analysis (GSEA) was conducted using R (version 4.3.3) to assess the biological significance of gene expression differences and identify enriched pathways in SCD, HFD, and dietary intervention groups. The analysis was performed using the clusterProfiler package in R (version 4.12.6), utilizing gene sets from the Hallmark and C2 collections within the Molecular Signatures Database (MSigDB). Gene expression data were first ranked by log fold change from the comparison groups, with pathways related to key processes such as platelet activation, inflammation, and mitochondrial function being the primary focus of the analysis.

The gene expression data were mapped to appropriate gene symbols using the Mouse Gene Symbol Remapping for Human Orthologs (MsigDB v2024.1.Hs.Chip) to ensure cross-species comparability in the dataset. GSEA was executed with parameters including an exponent of 1, a maximum gene set size (MaxGSSize) of 1000, and a minimum gene set size (MinGSSize) of 1. Pathways were considered significant if they achieved a q-value, which represents an adjusted *p*-value accounting for the false discovery rate (FDR), following adjustments for multiple testing. GSEA results were compared across experimental groups to identify pathways significantly enriched in the relevant biological processes of interest.

### 2.5. Data Visualization and Statistical Analysis

Data visualization was performed using ggplot2 (version 3.5.1) and forcats (version 1.0.0) packages in R. The results of GSEA were visualized using dot plots, which display the top upregulated and downregulated pathways, with the size of the dots representing the number of enriched genes and the GeneRatio indicating the proportion of enriched genes relative to the total number in each pathway. Enrichment plots were used to show the enrichment score (ES) for individual pathways, with the x-axis representing the ranked list of genes and the y-axis showing the ES across the groups. Ridge plots were used to compare the normalized enrichment score (NES) distributions for upregulated and downregulated pathways between different experimental groups. Additionally, upset plots were employed to illustrate the overlap of enriched genes between pathways showing the highest and lowest NES values, allowing for visualization of shared and distinct pathway enrichments across the experimental groups. Heatmaps were generated using MeV (Multiple Experiment Viewer) or ggplot2 in R to visualize gene expression levels across key pathways involved in platelet activation, inflammation, and mitochondrial function in the experimental groups. Network plots created using STRING (version 12.0) were used to visualize interactions among key genes, including interferon gamma (IFNG), TNF, and IL-6, which are critical in the regulation of inflammation and thrombosis. Pathway analysis was performed using pre-ranked GSEA in the clusterProfiler package, as detailed in Section 2.3. Pathways were considered significant if they achieved a q-value < 0.05, reflecting adjustments for multiple testing to control for the FDR.

## 3. Results

### 3.1. Allulose Reduces Platelet Activation and Aggregation Pathways in SCD and HFD Models

**Figure 1:**
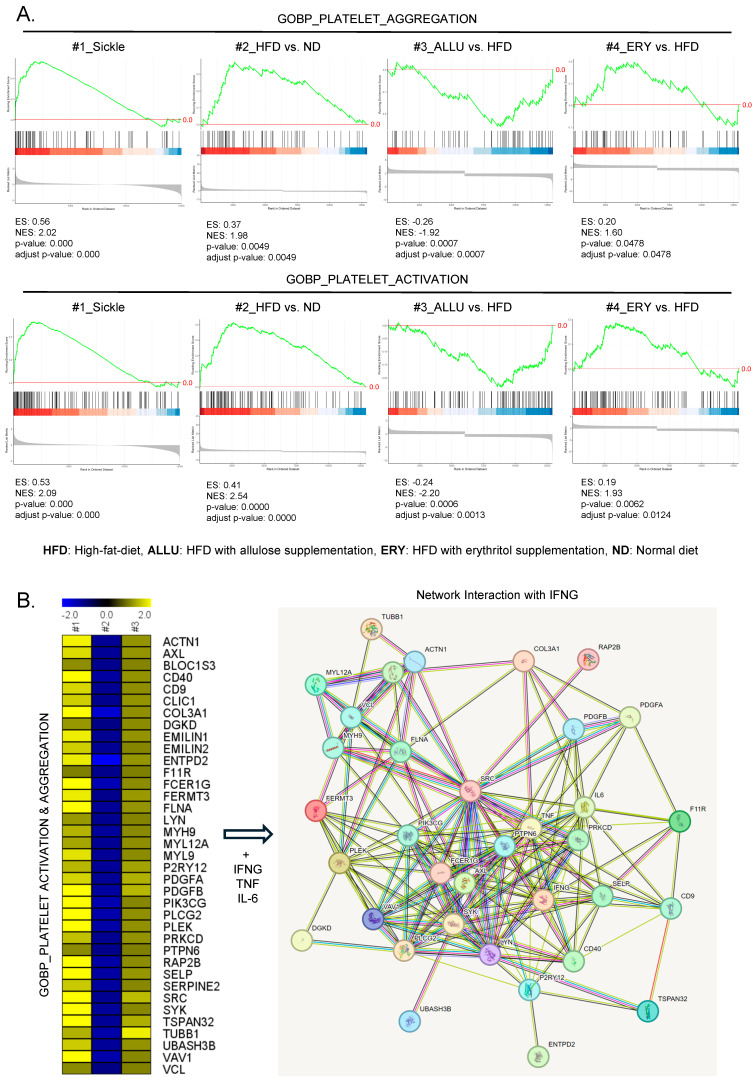
Comparison analysis of platelet activation and aggregation pathways in SCD patients and HFD-fed mice with or without allulose and erythritol. (**A**) GSEA enrichment plots for the platelet aggregation and activation pathways from the GOBP across the ALLU (HFD with allulose) and ERY (HFD with erythritol) groups compared to SCD patients. The ES quantifies the degree of gene expression enrichment, where positive values signify higher gene expression levels. (**B**) Heatmap illustrating the expression levels of genes enriched in platelet activation and aggregation pathways across three key comparisons: #1: HFD vs. ND (normal diet), #2: ALLU vs. HFD, and #3: ERY vs. HFD. The heatmap visualizes gene expression levels, and the accompanying network plot highlights key gene interactions involving IFNG, TNF, and IL-6, which play pivotal roles in regulating inflammation and thrombosis.

GSEA revealed distinct differences in the regulation of platelet aggregation and activation pathways among the SCD, HFD, and dietary intervention groups (allulose and erythritol). As shown in Figure 1A, platelet aggregation and activation pathways were significantly upregulated in both SCD and HFD with and without erythritol groups, indicating similar pro-thrombotic gene expression profiles. In contrast, the ALLU group exhibited a significant downregulation of these pathways, suggesting that allulose exerts an inhibitory effect on platelet activation. This is particularly important given the role of platelet activation in thrombotic complications associated with SCD. Each overlapping gene’s expression levels related to platelet aggregation and activation are shown in the heatmap in Supplemental Figure S1.

The heatmap in Figure 1B further illustrates this suppression, showing a distinct decrease in the expression of pro-thrombotic genes in the HFD group compared to ALLU and ERY groups. The network plot emphasizes the interaction between key inflammatory mediators such as IFNG, TNF, and IL-6, all of which are known to play crucial roles in inflammatory responses and thrombosis. The genes are dampened by ALLU group but remain active in both the HFD and ERY groups. This suggests that allulose may offer protective benefits by modulating these pathways, reducing the risk of thrombosis commonly associated with SCD and HFD conditions.

### 3.2. Differential Regulation of Top Upregulated or Downregulated SCD Pathways

**Figure 2:**
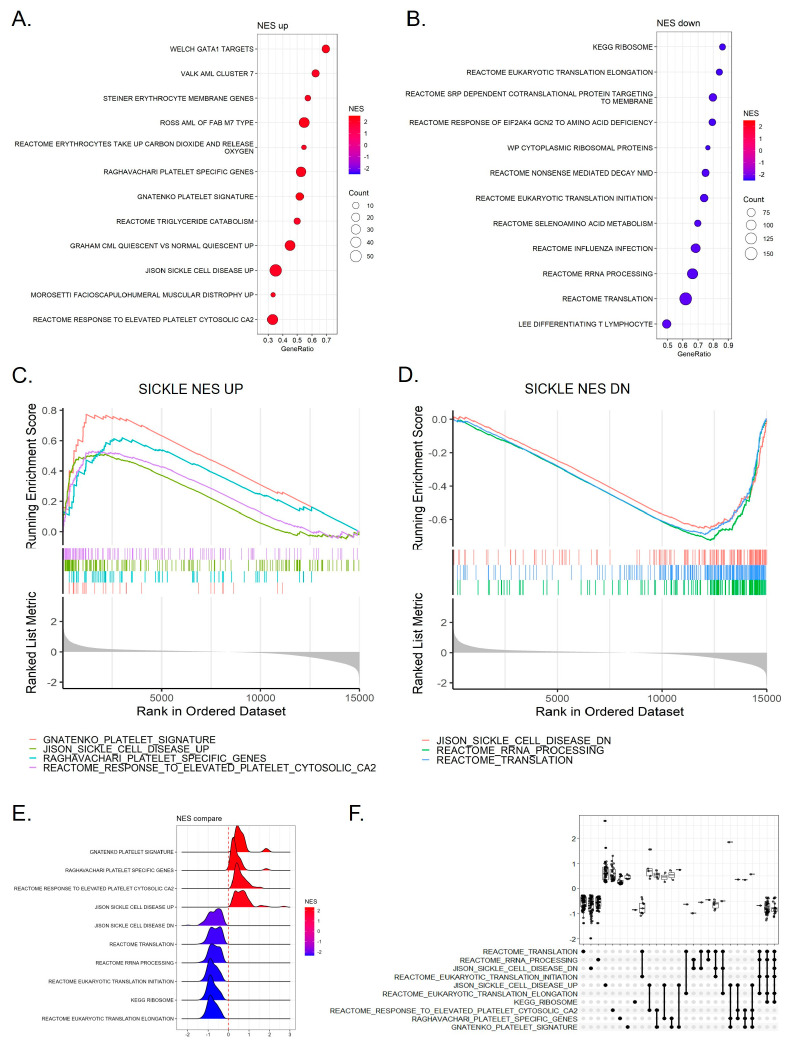
Top up- and downregulated pathways of platelet gene expression from SCD patients using C2 gene sets from MSigDB. (**A**,**B**) Dot plot showing the top 12 upregulated (**A**) and bottom 12 downregulated (**B**) pathways based on NES for platelet gene expression in SCD patients. Dot size represents the number of enriched genes, and the GeneRatio indicates the proportion of enriched genes within each pathway. (**C**,**D**) Enrichment plots visualizing the distribution of gene sets in the most significantly upregulated (**C**) and downregulated (**D**) pathways. The ES illustrates the concentration of platelet-specific genes at either end of the ranked gene list. (**E**) Ridge plot depicting the NES distribution between upregulated and downregulated pathways, allowing for a comparison of the magnitude of differential expression based on logFC values. (**F**) Upset plot showing the relationships between top and bottom NES pathways.

Figure 2 provides a comprehensive analysis of the top upregulated and downregulated pathways associated with platelet gene expression in SCD patients using C2 gene sets from MSigDB. This analysis helps to identify key biological processes impacted by the disease and highlights the regulatory differences in these pathways. The dot plot (Figure 2A) highlights the top upregulated pathways involved in SCD, focusing on platelet activation and pro-thrombotic mechanisms. Pathways such as GNATENKO platelet signature, Raghavachari platelet-specific genes, and Reactome response to elevated platelet cytosolic calcium were significantly upregulated, suggesting an elevated platelet activation response in SCD. The GeneRatio represents the fraction of genes in each pathway that are enriched relative to the total number of genes in that pathway, while the dot size indicates the number of genes involved in the enrichment. Larger dots and higher GeneRatios indicate stronger enrichment and involvement of more genes, suggesting a robust activation of these platelet-related pathways in SCD patients.

Conversely, the downregulated pathways (Figure 2B) primarily involve mitochondrial function and protein translation processes, such as Kyoto encyclopedia of genes and genomes (KEGG) ribosome, Reactome eukaryotic translation elongation, and Reactome SRP-dependent cotranslational protein targeting to membrane. These pathways are essential for maintaining cellular and mitochondrial function, and their suppression in SCD patients points toward mitochondrial dysfunction. Mitochondrial impairment is closely linked to energy production deficits and can affect the overall metabolic state, contributing to the disease pathology. The GSEA enrichment plots (Figure 2C,D) visually depict the distribution of gene sets across the ranked datasets. Pro-thrombotic pathways like GNATENKO platelet signature and sickle cell disease show enrichment on the positive end, emphasizing increased platelet activity in SCD (Figure 2C). Meanwhile, mitochondrial and protein translation pathways, including Reactome translation and rRNA processing, are enriched on the negative end, illustrating their downregulation and mitochondrial dysfunction in SCD patients (Figure 2D).

The ridge plot (Figure 2E) displays the distribution of NES for both upregulated and downregulated pathways, allowing for a direct comparison of the strength and extent of pathway enrichment. Platelet-related pathways show high NES values, confirming the hyperactivation of platelets in SCD. On the other hand, mitochondrial and translation pathways show lower NES scores, reflecting their suppression and indicating mitochondrial dysfunction in these patients. The upset plot (Figure 2F) emphasizes the overlap of enriched pathways between the top upregulated and downregulated pathways. It clearly shows that platelet activation-related pathways are upregulated in SCD patients, representing a key mechanism that drives thrombosis and platelet hyperactivity. In contrast, pathways related to mitochondrial translation are prominently downregulated in SCD patients, reflecting the significant mitochondrial dysfunction associated with the disease. This dual regulation highlights the critical role of both platelet activation and mitochondrial impairment in SCD pathophysiology, suggesting that targeting these pathways could be essential for therapeutic intervention.

### 3.3. Allulose Enhances Mitochondrial Function While Erythritol Mirrors SCD Pathways

**Figure 3:**
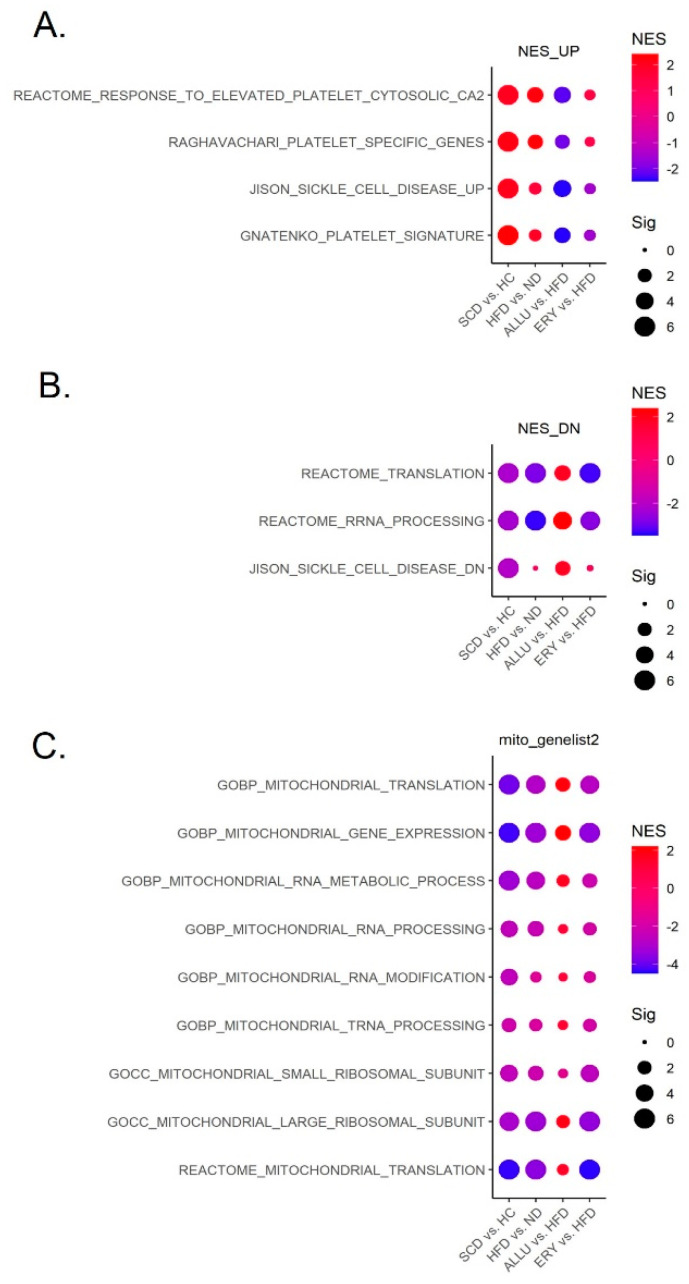
Modulation of SCD-altered gene sets by HFD with or without allulose and erythritol. (**A**) Dot plot showing gene sets upregulated in SCD and their modulation across HFD, ALLU, and ERY groups. The size of each dot represents the significance of the gene set, measured as −log(FDR q-value), while the color gradient from blue to red reflects the NES, where red indicates positive enrichment and blue indicates negative enrichment. (**B**) Dot plot displaying gene sets downregulated in SCD, with comparisons between the HFD, ALLU, and ERY groups. Dot size reflects significance (−log FDR q-value), and dot color indicates the NES, with red for positive and blue for negative enrichment. (**C**) Dot plot illustrating gene sets related to the mitochondrial translation process across the HFD, ALLU, and ERY groups. Dot size represents the significance (−log FDR q-value), and the color gradient shows NES, with red indicating positive enrichment and blue indicating negative enrichment.

Figure 3A displays the upregulated gene sets in SCD patients, with a comparison of their modulation across the HFD, ALLU, and ERY groups. The key pathways related to platelet activation, such as the Reactome response to elevated platelet cytosolic calcium and Raghavachari platelet-specific genes, are significantly upregulated in both SCD and HFD-fed mice. These results indicate that HFD exacerbates the platelet activation seen in SCD. However, ALLU treatment leads to a significant suppression of these pathways, suggesting that allulose mitigates the platelet hyperactivation caused by both HFD and SCD. In contrast, ERY did not exhibit a strong modulatory effect on these pathways, as the changes were not as prominent, indicating a lack of protective effect from erythritol.

In Figure 3B, the downregulated gene sets in SCD are displayed, focusing on pathways essential for mitochondrial protein synthesis and metabolic function, such as Reactome translation and rRNA processing. These pathways, which are downregulated in SCD, follow a similar pattern in the HFD group, demonstrating that SCD causes mitochondrial dysfunction similar to what is observed in obesity patients. However, the ALLU group shows a significant upregulation of these pathways, indicating a restoration of mitochondrial function under allulose treatment. This suggests that allulose plays a protective role by reversing the mitochondrial dysfunction associated with both SCD and HFD. Conversely, the ERY group shows a trend toward further suppression of these pathways, suggesting that erythritol may worsen mitochondrial dysfunction in these models. Each overlapping gene’s expression levels related to top up- and downregulated pathways in SCD patients are shown in the heatmap in Supplemental Figure S2.

Figure 3C provides a focused analysis on mitochondrial translation and related gene sets across the different groups. Gene sets such as Gene Ontology Biological Process (GOBP) mitochondrial RNA metabolic process, mitochondrial gene expression, and mitochondrial translation show negative enrichment in both the HFD and ERY groups, represented by blue dots. This indicates mitochondrial dysfunction in these groups, likely contributing to the overall metabolic dysregulation and increased risk of thrombotic events. Particularly, the SCD group exhibits a similar pattern to the HFD group, implying that both conditions share analogous pathological mechanisms, especially in terms of mitochondrial dysfunction. This highlights the potential for targeting similar pathways in SCD and HFD to alleviate these dysfunctions. In contrast, the ALLU group shows less negative enrichment, and in some cases, even positive modulation, suggesting that allulose helps mitigate mitochondrial dysfunction and promotes mitochondrial health. This improvement in mitochondrial translation and gene expression highlights allulose’s potential to restore mitochondrial function and counteract the negative effects observed with both HFD and SCD. Each overlapping gene’s expression levels related to REACTOME mitochondrial translation pathway in each group are shown in the heatmap in Supplemental Figure S3.

In summary, Figure 3 shows that allulose not only helps reduce platelet activation but also significantly improves mitochondrial function, reversing the mitochondrial dysfunction seen in both SCD and HFD-fed mice. On the other hand, erythritol tends to exacerbate both platelet activation and mitochondrial dysfunction, particularly in the HFD group, highlighting the distinct metabolic effects of these two sugar substitutes.

### 3.4. Allulose Improves Electron Transport Chain (ETC) Activity and Energy Metabolism Dysregulated in SCD

**Figure 4:**
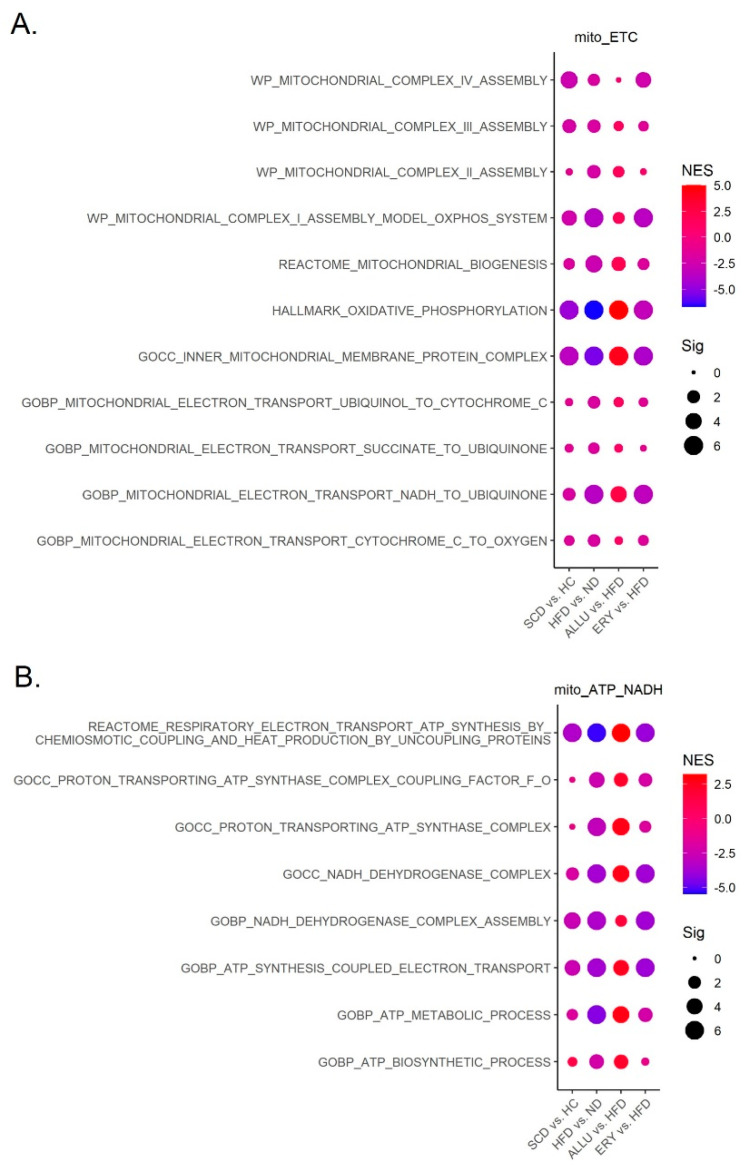
GSEA of mitochondrial-related pathways in SCD, HFD, ALLU, and ERY groups. (**A**) Dot plot illustrating the regulation of ETC pathways across the SCD, HFD, ALLU, and ERY groups. The size of each dot represents the significance of the pathway, measured as −log(FDR q-value), while the color gradient from blue to red reflects the NES. Red indicates positive enrichment and blue indicates negative enrichment. The ALLU group shows significant upregulation of ETC pathways compared to the SCD, HFD, and ERY groups. (**B**) Dot plot of pathways related to ATP synthesis and NADH metabolism in the SCD, HFD, ALLU, and ERY groups. Larger dots indicate higher significance (−log FDR q-value), while the color gradient shows the NES, with red representing positively enriched pathways and blue representing negatively enriched pathways. ALLU exhibits the most significant upregulation compared to the other groups.

Figure 4A illustrates that ETC and ATP synthesis pathways were significantly downregulated in both the HFD and ERY groups. These downregulations suggest mitochondrial dysfunction, with impaired energy metabolism and ATP production, contributing to the increased oxidative stress seen in these models. This pattern of mitochondrial dysfunction is also reflected in SCD, showing a similar tendency toward reduced mitochondrial activity and bioenergetics. In stark contrast, the ALLU group displayed marked upregulation of mitochondrial pathways, particularly those involved in ETC function and ATP production. The upregulation of these pathways suggests that allulose plays a critical role in improving mitochondrial bioenergetics, enhancing ATP synthesis, and supporting NADH metabolism, as seen in Figure 4B. The increased activity in these pathways indicates a more efficient energy metabolism, which helps to counter the mitochondrial dysfunction commonly observed in SCD and HFD-induced conditions. This restoration of mitochondrial function in the ALLU group implies that allulose may alleviate mitochondrial stress, reducing the platelet hyperactivation and thrombotic risks that are characteristic of SCD and other metabolic disorders. The ability of allulose to improve ATP production and mitigate oxidative stress could be a critical mechanism through which it modulates platelet function and reduces the pro-thrombotic environment seen in both SCD patients and HFD-fed mice. In summary, the enhanced ETC activity and energy metabolism observed with allulose treatment positions it as a promising candidate for reducing the metabolic and thrombotic complications associated with mitochondrial dysfunction in both SCD and HFD-induced disorders. Each overlapping gene’s expression levels in GOBP mitochondrial electron transport NADH to ubiquinone and GOBP ATP synthesis coupled electron transport pathway in each group are shown in the heatmap in Supplemental Figure S4.

## 4. Discussion

The findings of this study provide compelling evidence supporting the potential of allulose as a safer alternative to erythritol, particularly in individuals at heightened risk of thrombotic events, such as those with SCD or those consuming an HFD. The distinct mechanistic differences in how these sugar substitutes influence platelet activation and mitochondrial function could have far-reaching implications for dietary recommendations and the management of cardiometabolic health.

In our study, we demonstrated that erythritol, although widely used for its low caloric content and minimal effect on blood sugar levels, significantly exacerbated platelet activation and aggregation in HFD-fed mice. These findings align with previous research raising concerns about erythritol’s potential to promote thrombosis through increased platelet aggregation. Studies such as that by Witkowski et al. demonstrated that elevated circulating erythritol levels are linked to an increased risk of major adverse cardiovascular events (MACEs) in patients undergoing cardiac evaluation [2]. In both in vitro and in vivo studies, erythritol was shown to enhance platelet reactivity and promote thrombosis, raising concerns about its long-term safety. Notably, our findings add a new dimension by showing that erythritol consumption in HFD models can further amplify the platelet activation and aggregation already induced by HFD. This highlights the potential risks associated with erythritol consumption in individuals on HFD, suggesting that careful consideration is needed when recommending erythritol in such populations.

In contrast to erythritol, allulose demonstrated a significantly different effect by reducing platelet activation and aggregation pathways that are typically activated by an HFD. According to our previous research, we have shown that interferon-gamma (IFN-γ) plays a mechanistic role in initiating systemic inflammation in white adipose tissue via early signaling in the liver during HFD-induced chronic inflammation [13]. Although a more detailed mechanistic analysis of these physiological differences is forthcoming in another study, the current findings suggest that IFN-γ, along with IL-6 and TNF-α, are central to the platelet activation and aggregation pathways triggered by HFD. Furthermore, both IL-6 and TNF-α are cytokines closely associated with the pathogenesis of SCD, as they play critical roles in exacerbating inflammatory responses and promoting thrombotic complications in SCD patients [18]. The overlap between the inflammatory mechanisms seen in both HFD and SCD pathogenesis—particularly the involvement of these key cytokines—suggests a shared pathway that links systemic inflammation to platelet activation. This mechanistic convergence highlights the broader impact of allulose in modulating platelet function, not only in metabolic disorders like those induced by HFD but also in conditions characterized by heightened inflammation, such as SCD.

An essential finding of this study is the connection between mitochondrial dysfunction and platelet aggregation, highlighting its significance in both metabolic and hematologic disorders. Previous research has demonstrated that IFN-γ-induced mitochondrial dysfunction in macrophages plays a crucial role in systemic inflammation triggered by a HFD [13,19]. This mechanism, while primarily observed in macrophages, may extend to platelets, where mitochondrial dysfunction contributes to the pathogenesis of SCD. Mitochondrial dysfunction is strongly implicated in platelet aggregation and morphological changes [20,21], with recent studies showing that mitochondrial gene expression is necessary for platelet activation. Additionally, disruptions in RNA-binding proteins have been linked to thrombocytopenia and excessive bleeding, underscoring the central role of mitochondrial function in platelet biology [22].

Our study further reinforces these mechanistic insights, showing significant downregulation of mitochondrial translation pathways in SCD, consistent with previous reports linking mitochondrial dysfunction, particularly of mitochondrial complex I within the ETC, to platelet dysregulation and inflammation. Notably, our previous work demonstrated that abnormalities in mitochondrial complex I are highly correlated with inflammatory and metabolic dysfunction in HFD-induced conditions [8,13]. Similar mitochondrial deficiencies were observed in SCD patients, suggesting that mitochondrial dysfunction is a shared feature misleading platelet activity in both HFD-induced metabolic stress and SCD.

The potential benefits of allulose to SCD patients are particularly significant. SCD is characterized by chronic inflammation, oxidative stress, and a heightened risk of thrombotic events, making it essential to explore safe dietary interventions. Our findings demonstrated that allulose improves mitochondrial function, in contrast to erythritol, which exacerbates mitochondrial dysfunction. Given the similarities between the mechanisms driving systemic inflammation in HFD and those exacerbating SCD, allulose offers dual benefits: reducing HFD-induced inflammation and directly improving mitochondrial function in the platelets of SCD patients.

## 5. Conclusions

Overall, this study demonstrates the potential of allulose to offer significant antithrombotic benefits, particularly in high-risk populations such as those with SCD or those consuming an HFD. Unlike erythritol, which exacerbates pro-thrombotic pathways, allulose appears to modulate critical pathways associated with platelet function and mitochondrial activity, reducing inflammation and promoting mitochondrial health. These findings underscore the importance of considering not only the metabolic, but also the cardiovascular impacts of sugar substitutes, and suggest that allulose may be a preferable option in individuals at risk of thrombotic events. Further research, particularly in human clinical trials, is warranted to confirm these findings and explore the broader applications of allulose in managing cardiometabolic and thrombotic risks.

## Data Availability

The raw and processed RNA-sequencing data from the animal experiments in this study have been deposited in the Gene Expression Omnibus (GEO) under accession number GSE28013. Data pertaining to SCD patients were obtained from GEO accession number GSE11524, as described in the Methods section. Further details are available from the corresponding author upon request due to time limitations.

## Supplementary Materials

The following supporting information can be downloaded at: https://www.mdpi.com/article/10.3390/nu16244295/s1, Figure S1: Heatmap of comparison in platelet activation and aggregation pathways in SCD (sickle cell disease) patients and HFD (high-fat diet)-fed mice with or without allulose and erythritol. (A,B) Each cell in the heatmap represents the overlapping genes across the SCD, HFD, ALLU (HFD with allulose) and ERY (HFD with erythritol) groups that showed significant differences in the GSEA results of platelet aggregation and activation. The color range of cells is based on the log ratio. The range is set for presenting subtle variations in each pathway; Figure S2: Heatmap of comparison in top up- and downregulated pathways of platelet gene expression from SCD in SCD patients and HFD-fed mice with or without allulose and erythritol. (A–D) Each cell in the heatmap represents the overlapping genes across the SCD, HFD, ALLU and ERY groups that showed significant differences in the GSEA results of most upregulated pathways of platelet gene expression from SCD. (E–G) Each cell in the heatmap represents the overlapping genes in all groups that showed significant differences in the GSEA results of most downregulated pathways of platelet gene expression from SCD; Figure S3: Heatmap of comparison in the REACTOME mitochondrial translation pathway in SCD patients and HFD-fed mice with or without allulose and erythritol. Each cell in the heatmap represents the overlapping genes across the SCD, HFD, ALLU and ERY groups that showed significant differences in the GSEA results of REACTOME mitochondrial translation; Figure S4: Heatmap of comparison in mitochondrial-related pathway in SCD patients and HFD-fed mice with or without allulose and erythritol. (A) Each cell in the heatmap represents the overlapping genes across the SCD, HFD, ALLU and ERY groups that showed significant differences in the GSEA results of GOBP mitochondrial electron transport NADH to ubiquinone. (B) Each cell in the heatmap represents the overlapping genes across the SCD, HFD, ALLU and ERY groups that showed significant differences in the GSEA results of GOBP ATP synthesis coupled electron transport.
