# Supplementary material for: Pathway Analysis of Allulose as a Sugar Substitute in Mitigating Thrombotic Risks in Sickle Cell Disease Patients
Source: Nutrients. 2024 Dec 12;16(24):4295. doi: 10.3390/nu16244295 (PMC11678832; doi:10.3390/nu16244295)
Supplement: Supplementary file 1 [file nutrients-16-04295-s001.zip › nutrients-3268464-supplementary.pdf]

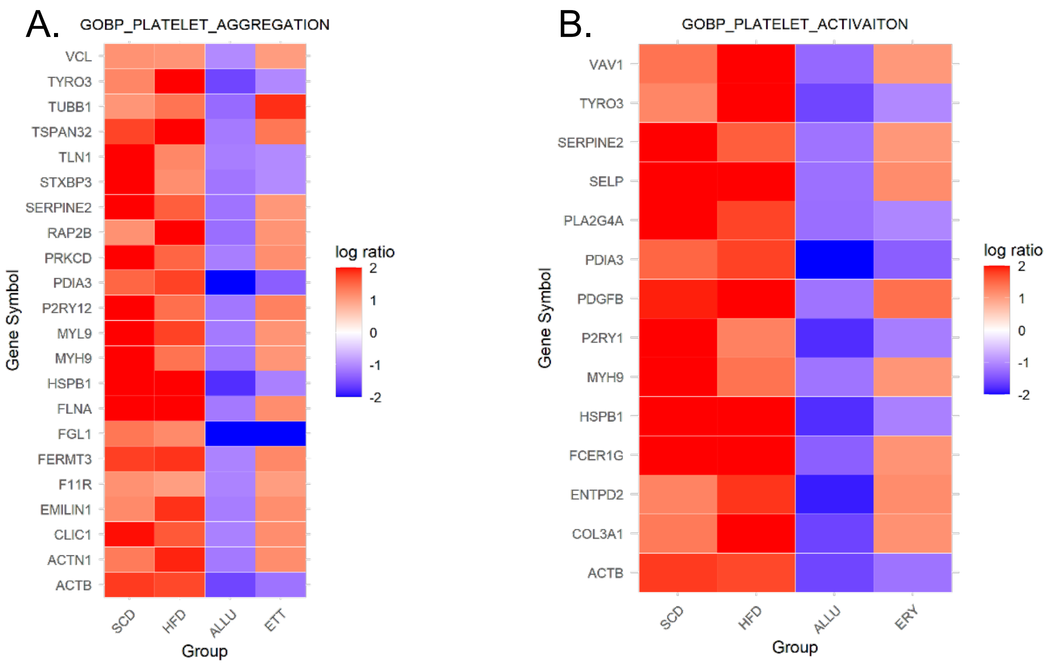

Supplementary Figure S1. Heatmap of comparison in platelet activation and aggregation pathways in SCD (Sickle cell disease) patients and HFD (High-fat diet)-fed mice with or without allulose and erythritol.

(A - B) Each cell in the heatmap represents the overlapping genes across the SCD, HFD, ALLU (HFD with allulose) and ERY (HFD with erythritol) groups that showed significant differences of the GSEA results of platelet aggregation and activation. The color range of cells are based on the log ratio. The range is set for presenting subtle variations in each pathway.

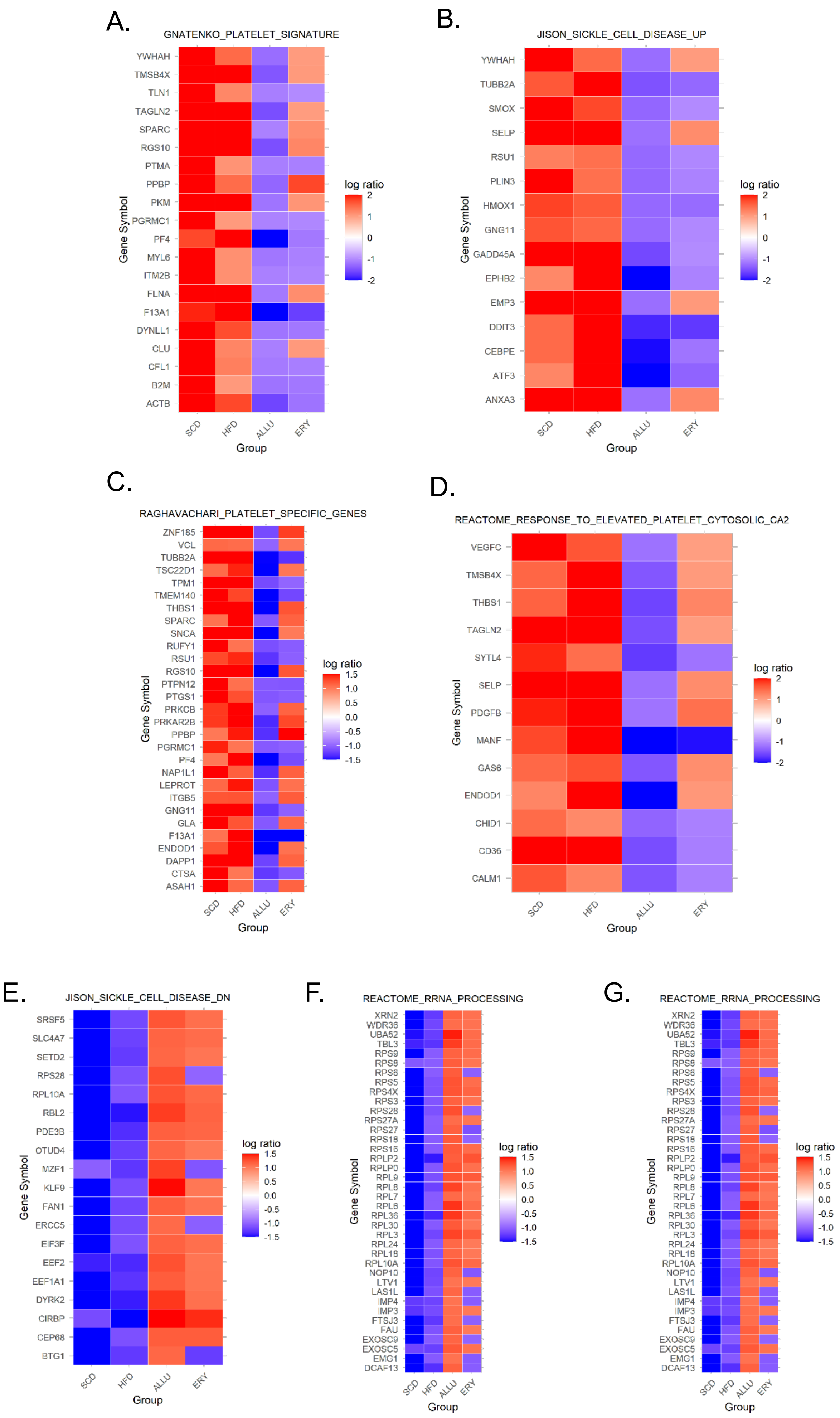
A.
GNATENKO_PLATELET_SIGNATURE
YWHAH
TMSB4X
TLN1
TAGLN2
SPARC
RGS10
PTMA
PPBP
PKM
PGRMC1
PF4
MYL6
ITM2B
FLNA
F13A1
DYNLL1
CLU
CFL1
B2M
ACTB
Gene Symbol
SCD
HFD
ALLU
ERY
Group
log ratio
2
1
0
-1
-2
B.
JISON_SICKLE_CELL_DISEASE_UP
YWHAH
TUBB2A
SMOX
SELP
RSU1
PLIN3
HMOX1
GNG11
GADD45A
EPHB2
EMP3
DDIT3
CEBPE
ATF3
ANXA3
Gene Symbol
SCD
HFD
ALLU
ERY
Group
log ratio
2
1
0
-1
-2
C.
RAGHAVACHARI_PLATELET_SPECIFIC_GENES
ZNF185
VCL
TUBB2A
TSC22D1
TPM1
TMEM140
THBS1
SPARC
SNCA
RUFY1
RSU1
RGS10
PTPN12
PTGS1
PRKCB
PRKAR2B
PPBP
PGRMC1
PF4
NAP1L1
LEPROT
ITGB5
GNG11
GLA
F13A1
ENDOD1
DAPP1
CTSA
ASAH1
Gene Symbol
SCD
HFD
ALLU
ERY
Group
log ratio
1.5
1.0
0.5
0.0
-0.5
-1.0
-1.5
D.
REACTOME_RESPONSE_TO_ELEVATED_PLATELET_CYTOSOLIC_CA2
VEGFC
TMSB4X
THBS1
TAGLN2
SYTL4
SELP
PDGFB
MANF
GAS6
ENDOD1
CHID1
CD36
CALM1
Gene Symbol
SCD
HFD
ALLU
ERY
Group
log ratio
2
1
0
-1
-2
E.
JISON_SICKLE_CELL_DISEASE_DN
SRSF5
SLC4A7
SETD2
RPS28
RPL10A
RBL2
PDE3B
OTUD4
MZF1
KLF9
FAN1
ERCC5
EIF3F
EEF2
EEF1A1
DYRK2
CIRBP
CEP68
BTG1
Gene Symbol
SCD
HFD
ALLU
ERY
Group
log ratio
1.5
1.0
0.5
0.0
-0.5
-1.0
-1.5
F.
REACTOME_RRNA_PROCESSING
XRN2
WDR36
UBA52
TBL3
RPS9
RPS8
RPS6
RPS5
RPS4X
RPS3
RPS28
RPS27A
RPS27
RPS18
RPS16
RPLP2
RPLP0
RPL9
RPL8
RPL7
RPL6
RPL36
RPL30
RPL3
RPL24
RPL18
RPL10A
NOP10
LTV1
LAS1L
IMP4
IMP3
FTSJ3
FAU
EXOSC9
EXOSC5
EMG1
DCAF13
Gene Symbol
SCD
HFD
ALLU
ERY
Group
log ratio
1.5
1.0
0.5
0.0
-0.5
-1.0
-1.5
G.
REACTOME_RRNA_PROCESSING
XRN2
WDR36
UBA52
TBL3
RPS9
RPS8
RPS6
RPS5
RPS4X
RPS3
RPS28
RPS27A
RPS27
RPS18
RPS16
RPLP2
RPLP0
RPL9
RPL8
RPL7
RPL6
RPL36
RPL30
RPL3
RPL24
RPL18
RPL10A
NOP10
LTV1
LAS1L
IMP4
IMP3
FTSJ3
FAU
EXOSC9
EXOSC5
EMG1
DCAF13
Gene Symbol
SCD
HFD
ALLU
ERY
Group
log ratio
1.5
1.0
0.5
0.0
-0.5
-1.0
-1.5

Supplementary Figure S2. Heatmap of comparison in Top up- and down-regulated pathways of platelet gene expression from SCD in SCD patients and HFD-fed mice with or without allulose and erythritol.

(A - D) Each cell in the heatmap represents the overlapping genes across the SCD, HFD, ALLU and ERY groups that showed significant differences of the GSEA results of most up-regulated pathways of platelet gene expression from SCD. (E-G) Each cell in the heatmap represents the overlapping genes in all groups that showed significant differences of the GSEA results of most down-regulated pathways of platelet gene expression from SCD.

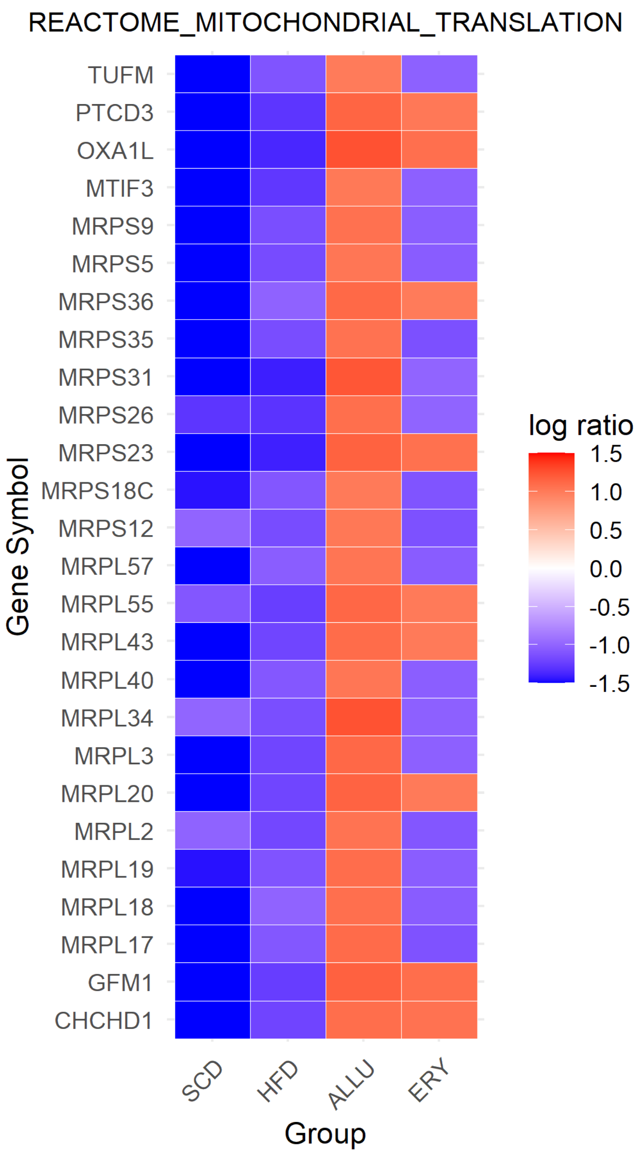

Supplementary Figure S3. Heatmap of comparison in the REACTOME mitochondrial translation pathway in SCD patients and HFD-fed mice with or without allulose and erythritol.

Each cell in the heatmap represents the overlapping genes across the SCD, HFD, ALLU and ERY groups that showed significant differences of the GSEA results of REACTOME mitochondrial translation.

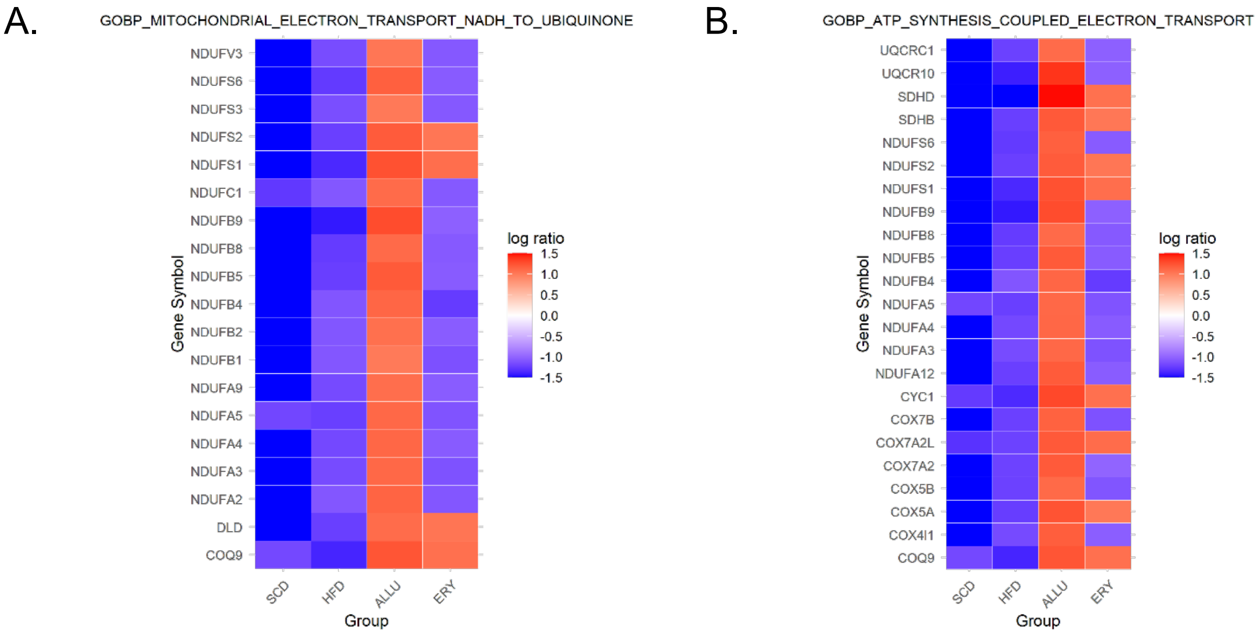

Supplementary Figure S4. Heatmap of comparison in mitochondrial-related pathway in SCD patients and HFD-fed mice with or without allulose and erythritol.

(A) Each cell in the heatmap represents the overlapping genes across the SCD, HFD, ALLU and ERY groups that showed significant differences of the GSEA results of GOBP mitochondrial electron transport NADH to ubiquinone. (B) Each cell in the heatmap represents the overlapping genes across the SCD, HFD, ALLU and ERY groups that showed significant differences of the GSEA results of GOBP ATP synthesis coupled electron transport.
